# Bioavailability Assessment of Heavy Metals Using Various Multi-Element Extractants in an Indigenous Zinc Smelting Contaminated Site, Southwestern China

**DOI:** 10.3390/ijerph18168560

**Published:** 2021-08-13

**Authors:** Jun-Xian Wang, Da-Mao Xu, Rong-Bing Fu, Jia-Peng Chen

**Affiliations:** 1 Centre for Environmental Risk Management and Remediation of Soil and Groundwater, Tongji University, Shanghai 200092, China; junxianwang@alumni.usc.edu; 2 Viterbi School of Engineering, University of Southern California, Los Angeles, CA 90089, USA; 3 State Key Laboratory of Pollution Control and Resources Reuse, College of Environmental Science and Engineering, Tongji University, Shanghai 200092, China; 1751804@tongji.edu.cn; 4 Shanghai Institute of Pollution Control and Ecological Security, Shanghai 200092, China

**Keywords:** indigenous smelting contaminated soils, heavy metals, bioavailability, fractionation, release kinetics

## Abstract

Despite recent studies have investigated the strong influences of smelting activities on heavy metal contamination in the soil environment, little studies have been conducted on the current information about the potential environmental risks posed by toxic heavy metals in smelting contaminated sites. In the present study, a combination of the bioavailability, speciation, and release kinetics of toxic heavy metals in the indigenous zinc smelting contaminated soil were reliably used as an effective tool to support site risk assessment. The bioavailability results revealed that the bioavailable metal concentrations were intrinsically dependent on the types of chemical extractants. Interestingly, 0.02 mol/L EDTA + 0.5 mol/L CH_3_COONH_4_ was found to be the best extractant, which extracted 30.21% of Cu, 31.54% of Mn, 2.39% of Ni and 28.89% of Zn, respectively. The sequential extraction results suggested that Cd, Pb, and Zn were the most mobile elements, which would pose the potential risks to the environment. The correlation of metal bioavailability with their fractionation implied that the exchangeable metal fractions were easily extracted by CaCl_2_ and Mehlich 1, while the carbonate and organic bound metal fractions could be extracted by EDTA and DTPA with stronger chelating ability. Moreover, the kinetic modeling results suggested that the chemical desorption mechanism might be the major factor controlling heavy metal release. These results could provide some valuable references for the risk assessment and management of heavy metals in the smelting contaminated sites.

## 1. Introduction

Heavy metal contamination around mining and smelting sites have received global concern [1,2]. Many studies have indicated that about 40–73% of toxic heavy metals emitted into the soil are closely associated with smelting activities [3,4]. Furthermore, many studies have reported that toxic heavy metals, such as, Cd, Pb, and Zn, have posed the potential risks to the ecosystem and human health around the smelting areas [5,6]. As a consequence, the risk assessment of heavy metal pollution in the smelting contaminated sites is an important task and challenge for the sustainable development of non-ferrous metal smelting industry [7,8]. It is well acknowledged that heavy metal toxicity depends on their geochemical fractions based on the bioavailability and sequential extraction method instead of their total contents in soils [9,10,11]. The previous studies are mostly concentrated on the total metal concentrations in soil near smelters to assess the potential risks resulted from excessive accumulation of heavy metals in agricultural crops, which could not accurately represent the part that can be absorbed and utilized by plants [12,13]. Meanwhile, the accurate evaluation for the potential health risks of heavy metals in contaminated soils largely depends on how precisely predicting the uptake concentrations of soil heavy metals by plants [14]. Moreover, although various chemical extraction methods have been developed to estimate the metal bioavailability in soils, the current knowledge of the bioavailability of heavy metals in smelting contaminated soils is still limited [15,16]. In this context, combining sequential extraction experiments and single extraction method with the release kinetics of heavy metals is a critical procedure to provide a better understanding of the geochemical speciation of heavy metals that influences the bioavailability and release behaviors of heavy metals in the smelting contaminated sites.

Based on the polluted soils by toxic heavy metal collected from a typical zinc smelting site, the aim of the present study was to (i) Obtain the information about the bioavailable metal fraction using various multi-element extractants; (ii) study the geochemical fractionation of heavy metals; (iii) investigate the release kinetics of heavy metals. The present study results would presumably provide an in-depth knowledge of the geochemical metal fraction, and also provide inspiration for developing the optimal remediation strategies to mitigate the potential environmental risks caused by heavy metals in smelting sites.

## 2. Materials and Methods

### 2.1. Site Description

In historical period, large-scale zinc smelting activities were carried out in northwestern Guizhou Province, China, especially Hezhang city and Weining county [17,18]. Weining county is one of the most typical indigenous zinc smelting contaminated site in China. A detailed description of the zinc smelting contaminated site was provided in the previous study reported by Xu et al. [17]. In Guizhou Province, Sulfide ore as sphalerite (ZnS) and carbonate Zn ore as smithsonite (ZnCO_3_) was the two major types of Zn ores used in the artisanal zinc smelting [19]. The basic principle of indigenous zinc smelting here is shown as follows: the zinc bearing minerals undergoes a redox reaction and is reduced to zero-valent zinc by gaining electrons during the combustion process. The main redox reactions are shown below [20]:

2ZnS + 3C + 5O_2_ = 2Zn + 3CO_2_ + 5SO_2_ (1)

2ZnCO_3_ + C = 2Zn + 3CO_2_ (2)

Due to the smelting treatment process is simple and not advanced, and thus the recovery rate for Zn is relatively low, and other associated metals such as Cu, Cd, and Cr are almost left in the smelting residue [21]. In addition, since toxic elements such as Zn, Pb, Cu, As, Cd, and Cr have their respective melting and boiling points, some of which have be entered into the surrounding soils via dry and wet deposition. Although the local zinc smelting activities were completely ceased in 2004, the activities have resulted in severe soil contamination with heavy metals [22,23].

### 2.2. Soil Sampling and Preparation

In the present study, a soil sample (104°20′40.61″ E, 26°57′22.06″ N) at a depth of 0–20 cm was collected from an indigenous zinc smelting contaminated site in Weining county. After being transported to the laboratory, the soil sample was air dried, ground, and then passed through a 0.15-mm nylon sieve. The soil sample were stored before analysis.

### 2.3. The Determination of Total Metal Concentration

About 0.2 g of the prepared sample were digested with a mixture of concentrated HCl (9 mL)- HNO_3_ (3 mL)-HF (5 mL) in a hot plate. After the digestion, the solution was cooled to room temperature, and then diluted with deionized water to 50 mL. The heavy metal (Al, As, Cd, Cr, Cu, Fe, Mn, Ni, Pb, and Zn) concentrations in the digestions were determined by ICP-OES (Agilent 720, Agilent Technologies, Santa Clara, CA, USA).

### 2.4. Soil Bioavailable Metal Extraction

A summary of the universal extractants used for the determination of available heavy metals in soils is shown in Table 1. In this study, seven chemical extractants were applied to determine the available metal concentrations in the smelting contaminated soils. In brief, about 2.0 g of dried soil was mixed with 20 mL of different extraction solution at a ratio of 1:10 (m/V), followed by shaking at 25 °C and 200 rpm for 4 h. The supernatants was centrifuged at 4000 rpm for 20 min, filtered through 0.45 μm filters, and then transferred to a 15 mL centrifuge. Subsequently, the filtered supernatants were stored at 4 °C until the available metal concentrations was determined by ICP-OES.

The preliminary extraction results of available multi-elements indicated that AA-EDTA extractant was found to be the best extractants to determine the available concentrations of toxic heavy metals in the studied soil sample (the extraction results of available metals were further shown in Results and Discussion Section). To study the release kinetics of available heavy metals in the smelting contaminated soil, about 2.0 g of soils was accurately weighed, mixed with 20 mL of AA-EDTA extraction solution, and shaken at 200 rpm/min at 25 °C. The extraction solution was collected at different contact times (0.5, 1, 2, 4, 8, 12, 24, 48, 72, and 96 h). The resulting suspension was centrifuged, filtered, and analyzed for heavy metal concentrations by ICP-OES, as the above batch experiment.

### 2.5. Speciation Distribution of Heavy Metals

The heavy metal fractionation in soils was determined using the Tessier sequential extraction method [26], which were distinct five fractions: F1: exchangeable, F2: carbonate-bound, F3: Fe-Mn oxides bound, F4: organic-sulfide bound, and F5: residual fractions.

### 2.6. Quality Assurance and Data Analyses

All experiments were measured in the three analytical replicates. The element recoveries in the standard soil samples (GBW07451) ranged from 88.92 to 114.41%. In addition, the accuracy of heavy metal fraction determination was evaluated by comparing the total metal content and the sum of their five fractions (F1:F5) in the soil sample, and the reliability of all the measured metal data were within 10% of relative standard deviation (RSD). The soil metal data were presented as mean ± standard deviation (SD) using Microsoft Excel 2010. Significant differences between different chemical extractant data were conducted using Tukey’s honestly significant difference (HSD) test (one-way ANOVA, *p* < 0.05) using OriginPro 2021. The relationship between the bioavailable fraction of heavy metals and their five fractions was conducted using OriginPro 2021. Other figures used in this study were drawn by OriginPro 2017.

## 3. Results and Discussion

### 3.1. Soil Metal Concentration

The total and bioavailable concentrations of heavy metals is presented in Table 2. As shown in Table 2, the studied heavy metals in the soil sample digested by HCl + HNO_3_ + HF followed the mean concentration order as: Fe > Al > Zn > Mn > Pb > Cu > Ni > As > Cd. Interestingly, the total concentrations of As (35.98 ± 6.84 mg/kg), Cd (5.28 ± 0.39 mg/kg), Pb (168.67 ± 10.10 mg/kg) and Zn (645.81 ± 34.95 mg/kg) were higher than the risk screening values (RSV, 6.5 < pH ≤ 7.5) for the soil contamination of agricultural soils, based on the Soil Environmental Quality (GB 15618-2018) [27]. The results indicated that the heavy metal enrichment was mainly caused by the long-term smelting activities. Similar results were reported for smelting contaminated soils. For instance, Lee et al. indicated that the concentrations of Zn, Pb, Cd, and Cu in soils near a large Zn smelter displayed a significant decrease with depth, and these smelter-derived metals were vertically migrated to 60 cm below the surface soils during the past 46 year zinc smelting [28]. Amnai et al. reported that the highest concentrations of Co, Cu, Fe, Mn, Ni, and Zn in the topsoils from slag heaps in an ancient iron smelting site were found up to 60, 20, 130,000, 8000, 60, and 250 mg/kg, respectively [29]. Li et al. found that As, Cd, Pb, and Zn in ground dust from a typical Chinese indigenous zinc smelting area were about ten times greater than those in other non-smelting cities [30]. Similarly, a recent study by Xu et al. also reported that the concentrations of Cu, Zn, As, Cd, and Pb in more than 50% of the selected non-ferrous metal smelting areas in China were higher RSV [31].

### 3.2. Soil Bioavailable Metal Fraction

**Figure 1:**
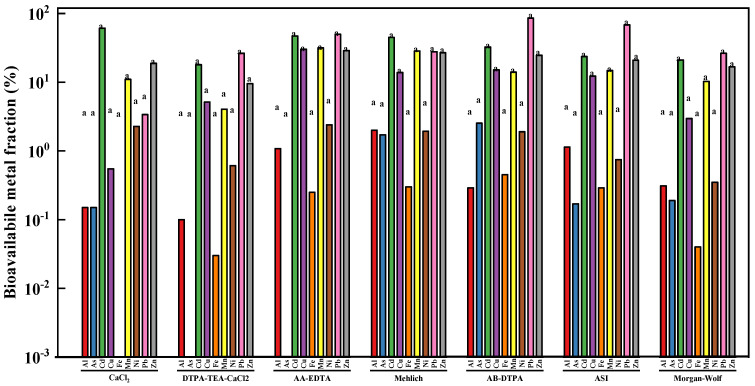
The mean bioavailable fraction (%) of toxic heavy metals in the studied soil sample using different chemical extractants [Note: Across a bar, values with different letters are significantly different (Tukey’s test *p* < 0.05)].

As shown in Figure 1 significant (*p* < 0.05) differences in bioavailable metal concentration were not found among seven chemical extractants. The bioavailable fractions of heavy metals in the tested soil sample are shown in Figure 1. AA-EDTA extracted the highest bioavailable concentrations of Cu (30.21 %), Mn (31.54 %), Ni (2.39 %), and Zn (28.89 %), while the AB-DTPA exhibited the greatest As (2.53 %) and Pb (85.73 %) concentrations. The bioavailable Cd concentration extracted by seven different extractants from the studied soil sample was in the descending order of CaCl_2_ > AA-EDTA > Mehlich I > AB-DTPA > ASI > Morgan-Wolf > DTPA-TEA-CaCl_2_. In addition, 2.00% of the total Al was extracted by Mehlich I from the studied soil, whereas CaCl_2_ only extracted 0.15% of Al.

Based on the above results, the significant differences in extraction ability among seven different extractants were fully reflected by their extractabilities in terms of bioavailable metal fraction. In addition, heavy metal solubility in different extractants varied markedly with soil physicochemical properties, including pH, organic matter (OM), and cation exchange capacity (CEC) [32,33]. In general, much higher concentration of Cu, Mn, Ni, and Zn was extracted by chelating agents including EDTA and DTPA from the studied soil sample, in comparison to the weaker extractants like CaCl_2_ and Mehlich I. It should be also noted that AA-EDTA was found to show the higher Cd, Cu, Mn, Ni, and Zn extractability than the other chelating extractants such as DTPA-TEA-CaCl_2_, AB-DTPA, ASI, and Morgan-Wolf, which were mainly due to the fact that AA-EDTA had higher concentration of EDTA (0.02 mol/L). The bioavailability results were consistent with many previous studies. For example, a previous study reported by Li et al. suggested that DTPA and CH_3_COONH_4_ extracted more metals from different types of heavy metal polluted soils, whereas water and NH_4_NO_3_ extracted a small amount of metals [34]. Another similar work carried out by Golui et al. indicated that EDTA extracted the highest extractability for heavy metals in the sludge-amended soil, followed by DTPA and CaCl_2_ [35].

### 3.3. Soil Metal Fractionation

**Figure 2:**
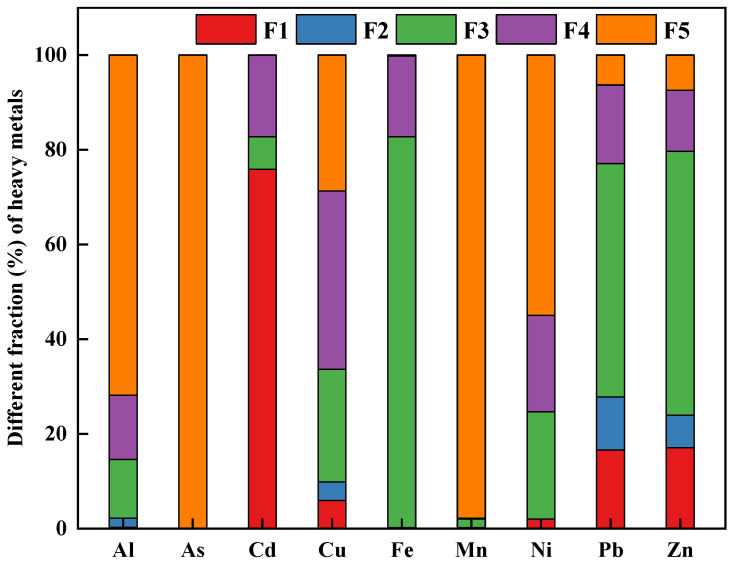
The fractionation of heavy metals in the studied soil sample.

Figure 2 illustrates the chemical speciation of heavy metals in the soil sample. The results from Tessier’ sequential extraction showed that Al (71.81%), As (100%), Mn (97.82%), and Ni (54.97%) were primarily bound to the residual fractions. Fe (82.53%), Pb (49.28%), and Zn (55.72%) were dominantly presented as the Fe-Mn oxide fraction. Previous studies have revealed that the Fe-Mn oxide fraction was an important part of total heavy metals, due to the adsorption and co-precipitation of trace metals with Fe/Mn oxide or hydroxide precipitation [36,37,38]. These results were consistent with our recent study, which indicated that sulfide oxidation and carbonate dissolution were the primary release mechanism of Fe, Pb, and Zn in the zinc-smelting slags [17]. Besides that, Pb and Zn showed the higher concentrations in the soil sample. It was meanwhile noted that the bioavailable fraction of Pb and Zn were relatively higher than other metals. Therefore, the potential environmental risks posed by Pb and Zn for local residents needed to be paid more attention. In addition, a large fraction of Cd (61.11 %) occurred in the exchangeable fraction. The finding indicated that among the studied metals, Cd was the most mobile element, which was further confirmed by the greatest Cd bioavailability extracted by CaCl_2_ solution. In case of Cu, the Fe-Mn oxides bound, organic-sulfide bound, and residual fraction accounted for 23.79, 37.72, and 28.66%, respectively. The results were similar to the previous studies, which implied that Cu showed a strong affinity to soil organic carbon [39,40]. Based on the sequential extraction results, the potential mobility [MF (%) =(F1 + F2)/(F1 + F2 + F3 + F4 + F5)] of the studied heavy metals were 2.16 % for Al, 0 % for As, 76.01 % for Cd, 0.27 % for Cr, 9.83 % for Cu, 0.20 % for Fe, 0.23 % for Mn, 2.05 % for Ni, 27.80 % for Pb, and 23.97 % for Zn, respectively. The above results suggested that Cd, Pb, and Zn were identified as the riskiest elements.

### 3.4. Impact of Heavy Metals Speciations on Their Bioavailability

**Figure 3:**
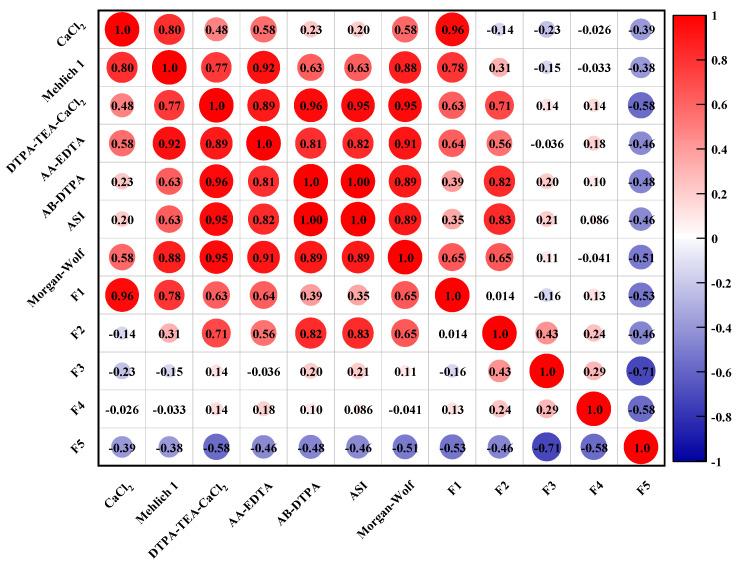
The relationship between the bioavailable fraction of heavy metals and their five fractions in the studied soil sample.

The correlations of heavy metals in the five fraction with their bioavailability are described in Figure 3. As shown in Figure 3, there are great differences in the correlation of the bioavailable fraction of toxic heavy metals and their respective five fractions in the soil sample. The significant correlation was found between the exchangeable fraction (F1) of heavy metals and their CaCl_2_ and Mehlich 1 extracted fractions. There are significant correlations of metal fractions extracted by DTPA-TEA-CaCl_2_, AA-EDTA, AB-DTPA, and ASI with their fractions in the carbonate-bound phases (F2). In addition, the correlation of the heavy metals presented as the organic-sulfide bound fraction (F4) with their AA-EDTA bioavailable fractions was found to be also positive. By combining these results from the correlation of metal bioavailability with their fractionation, it could be found that the metal species present in the exchangeable fraction were easily extracted by neutral salt and acid solution, while EDTA and DTPA with stronger chelating ability were able to extract metals from carbonate and organic bound pools, which was relatively difficult to release owing to the stronger ionic bonding.

### 3.5. Soil Metal Release Kinetics

**Figure 4:**
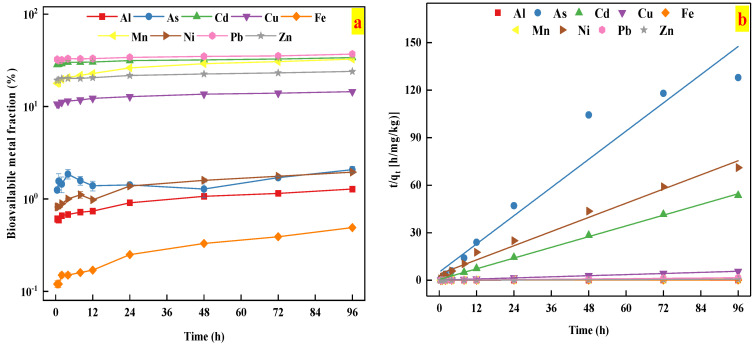
(**a**) The time-dependent bioavailability of heavy metals in the studied soil; (**b**) the kinetics of heavy metals release described by the second order model.

The release kinetics of heavy metals were better fitted by the second-order model than other kinetics models, as indicated by the high R^2^ values (R^2^ > 0.90), as shown in Table 3 and Figure 4b. This result indicated that chemical desorption mechanism might occur during the metal release from the studied soil [41,42].

The kinetics of heavy metal release from the studied soil sample is presented in Figure 4. As shown in Figure 4, the concentration of Cd, Cu, Mn, Pb, and Zn release from the studied soil tended to be relatively stable for 0.5–96 h, whose bioavailable fractions were in the range of 28.39–33.88%, 10.62–14.48%, 17.81–32.55%, 31.20–36.93%, and 19.24–24.01%, respectively. The finding implied that Cd, Cu, Mn, Pb, and Zn reached the release equilibrium within a short time period. In contrast, the concentration of bioavailable Al (0.59–1.28%), Fe (0.12–0.49%), and Ni (0.82–1.95%) indicated two distinct stages: a rapid increase in the initial stages (0–24 h), followed by a slow increase (24–96 h). Unlike other studied metals, the bioavailable fraction of As ranged from 1.25 to 2.08%, and showed three distinct stages: a rapid increase in the initial stages (0–4 h), a rapid decrease in the second stage (4–48 h), and reaching equilibrium (24–96 h). These results could be explained by the fact that as the release reaction progresses, the most bioavailable fractions of heavy metals was gradually released in the initial stages, and it took longer for these studied metal release to further contact the AA-EDTA extractant. These results were further confirmed by the results from soil metal bioavailability and fractionation tests.

## 4. Conclusions and Environmental Implication

In this work, notable differences (*p* < 0.05) were not found in the bioavailability of heavy metals determined by seven chemical extractants in the indigenous zinc smelting contaminated soil. However, the significant differences in the bioavailable concentration of toxic heavy metals had strong dependence on the types of different extractant solution. The bioavailable fractions of Cu, Mn, Ni, and Zn extracted by AA-EDTA were much higher than the other extractants. Moreover, Al, As, Mn, and Ni were present in a stable residual fraction. Instead, the higher concentrations of Cd, Pb, and Zn were found in the labile fractions, suggesting that these studied metals might cause a great risk to the environment. It should be also noted that neutral salt and acid solution easily extracted the exchangeable metal fraction, while EDTA and DTPA with stronger chelating ability could extract the carbonate and organic bound metal fractions. Furthermore, the release behaviors of heavy metals from the smelting contaminated soil was best fitted using the pseudo-second-order kinetic model, indicating that chemical desorption mechanism played an important role in the release of toxic heavy metals.

Based on the present study results, it is of greater importance to evaluate the potential environmental risks of toxic heavy metals in the smelting-contaminated sites using integrated geochemical and mineralogical strategies in the future. Moreover, it should be pointed out that the multi-disciplinary approaches and effective remediation measures need to be carried out for the better risk management and control of similar smelting contaminated sites throughout China.

## Figures and Tables

**Table 1:** Soil extraction methods proposed for extracting elements from soil samples [24,25].

Extractants	Chemical Reagents	pH	Soil Types	The Extracted Elements
Calcium chloride	0.1 mol/L CaCl_2_	-	-	Al, P, As, K, Mg, Ge, Na, Ni, B, Cu, Fe and Pb
Mehlich 1	0.05 mol/L HCl + 0.025 mol/L H_2_SO_4_	2.5	Neutral to alkaline soils	P, K, Ca, Mg, Na, Mn and Zn
DTPA-TEA-CaCl_2_	0.005 mol/L DTPA +0.1 mol/L TEA + 0.01 mol/LCaCl_2_	7.3	Calcareous soils	Cu, Fe, Mn and Zn
AA-EDTA	0.02 mol/L EDTA +0.5 mol/L CH_3_COONH_4_	4.65	Acidic soils	Cu, Fe, Mn and Zn
ASI	0.25 mol/L NaHCO_3_ + 0.01 mol/L EDTA + 0.01 mol/L NH4F	-	Acid to alkaline soils	P, K, Cu, Fe, Mn and Zn
AB-DTPA	1 mol/L NH_4_HCO_3_ + 0.005 mol/L DTPA	7.6	Alkaline soils	P, K, Na, Mn, Zn, As and Cd
Morgan-Wolf	0.073 mol/L CH_3_COONa + 0.52 mol/L CH_3_COOH + 0.0001 mol/L DTPA	4.8	Acidic soils	Al, P, As, K, Ca, Mg, B, Cu, Fe, Mn and Zn

**Table 2:** The total and bioavailable metal concentrations (mg/kg) in the studied soil sample.

Extractants		Elements	Al	As	Cd	Cu	Fe	Mn	Ni	Pb	Zn
Total concentration	HCl + HNO_3_ + HF	Mean	69,967.68	35.98	5.28	119.37	11,0511.76	402.07	69.35	168.67	645.81
		SD	4310.95	6.84	0.39	6.67	3572.14	15.11	2.86	10.10	34.95
Bioavailable concentrations	CaCl2	Mean	105.26	0.05	3.23	0.66	0.11	44.35	1.57	5.70	121.57
		SD	10.67	0.02	0.24	0.00	0.01	3.01	0.16	0.03	11.29
	DTPA-TEA-CaCl_2_	Mean	67.58	ND	0.95	6.14	33.28	16.26	0.42	44.49	61.35
		SD	1.46	ND	0.01	0.57	1.22	1.51	0.01	2.54	5.20
	AA-EDTA	Mean	756.36	ND	2.49	36.06	271.29	126.81	1.66	84.34	186.55
		SD	23.34	ND	0.05	0.43	8.56	1.16	0.02	1.38	2.49
	Mehlich 1	Mean	1398.35	0.62	2.37	16.48	329.20	114.01	1.34	46.75	173.45
		SD	26.22	0.03	0.08	0.17	7.52	3.02	0.01	0.75	1.66
	AB-DTPA	Mean	200.43	0.91	1.72	18.10	500.05	56.48	1.32	144.60	158.86
		SD	9.13	0.01	0.05	0.55	6.60	1.68	0.06	3.50	2.50
	ASI	Mean	799.14	0.06	1.25	14.62	321.65	59.43	0.52	115.13	134.65
		SD	1.69	0.03	0.01	0.16	6.63	0.68	0.10	1.04	1.08
	Morgan-Wolf	Mean	214.12	0.07	1.11	3.53	43.11	41.33	0.24	44.66	108.38
		SD	3.22	0.04	0.04	0.06	0.05	0.32	0.01	2.13	1.46

Note: ND represents concentration values were lower than the detection limit of the ICP-OES.

**Table 3:** The release kinetics model parameters of heavy metals in the studied soil sample.

Elements	Second Order Model	R_2_	SE	Constants	
				q_e_	k
Al	$\frac{t}{q_{t}}=\frac{1}{2.49\times 10^{-4}\times 884.95^{2}}+\frac{t}{884.95}$	0.986	1.69 × 10^−4^	884.95	2.49 × 10^−4^
As	$\frac{t}{q_{t}}=\frac{1}{0.43\times 0.67^{2}}+\frac{t}{0.67}$	0.937	1343.59	0.67	0.43
Cd	$\frac{t}{q_{t}}=\frac{1}{0.70\times 1.77^{2}}+\frac{t}{1.77}$	0.999	2.72	1.77	0.70
Cu	$\frac{t}{q_{t}}=\frac{1}{0.043\times 17.19^{2}}+\frac{t}{17.19}$	0.999	0.045	17.19	0.043
Fe	$\frac{t}{q_{t}}=\frac{1}{1.64\times 10^{-4}\times 531.91^{2}}+\frac{t}{531.91}$	0.919	0.0028	531.91	1.64 × 10^−4^
Mn	$\frac{t}{q_{t}}=\frac{1}{2.67\times 10^{-3}\times 130.28^{2}}+\frac{t}{130.28}$	0.995	0.0027	130.38	2.67 × 10^−3^
Ni	$\frac{t}{q_{t}}=\frac{1}{0.14\times 1.34^{2}}+\frac{t}{1.34}$	0.982	96.05	1.34	0.14
Pb	$\frac{t}{q_{t}}=\frac{1}{0.020\times 61.46^{2}}+\frac{t}{61.46}$	0.999	0.0027	61.46	0.020
Zn	$\frac{t}{q_{t}}=\frac{1}{5.81\times 10^{-3}\times 153.61^{2}}+\frac{t}{153.61}$	0.999	5.30 × 10^−4^	153.61	5.81 × 10^−3^

Note: q_t_ and q_e_ are the amount of metal released (mg/kg) from soil at time t and equilibrium; k is the rate constant [mg·(kg·min)^−1^], and t is time in h.

## Data Availability

All data generated or analyzed during this study are included in this published article.
